# An Endomyocardial Biopsy of the Left Ventricle in an Anorexia Nervosa Patient with Sinus Bradycardia and Left Ventricular Systolic Dysfunction

**DOI:** 10.1155/2016/9805291

**Published:** 2016-10-19

**Authors:** Satoshi Takahashi, Takanao Mine

**Affiliations:** Department of Internal Medicine, Cardiovascular Division, Hyogo College of Medicine, Nishinomiya, Japan

## Abstract

Anorexia nervosa (AN) is an eating disorder characterized by an abnormally low body weight, an intense fear of gaining weight, and a distorted perception of body weight. AN is a life-threatening condition that significantly increases the risk of death due to cardiac complications, such that at least one-third of all deaths in patients with AN are associated with cardiac causes including sudden death. In many reports, sudden death has been linked to reduced left ventricular function, structural changes, and QT abnormalities. However, the mechanistic details connecting AN to cardiac abnormalities remain unknown. Here we present an endomyocardial biopsy of the left ventricle in a case of AN with a reversible left ventricular systolic dysfunction.

## 1. Case Presentation

**Figure 1:**
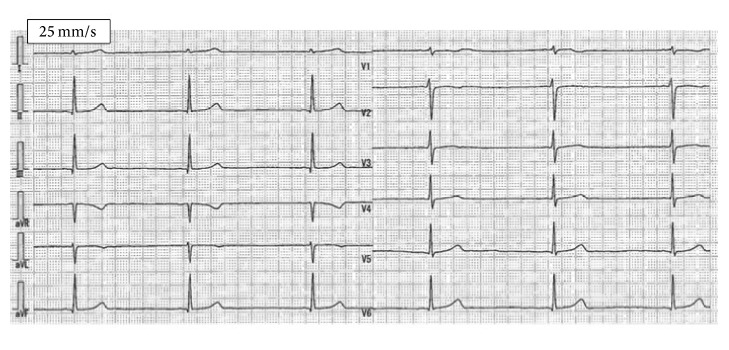
12-lead electrocardiogram shows bradycardia (heart rate 35 beats/min) with junctional rhythm; QT interval 520 ms (QTc interval 397 ms).

**Figure 2:**
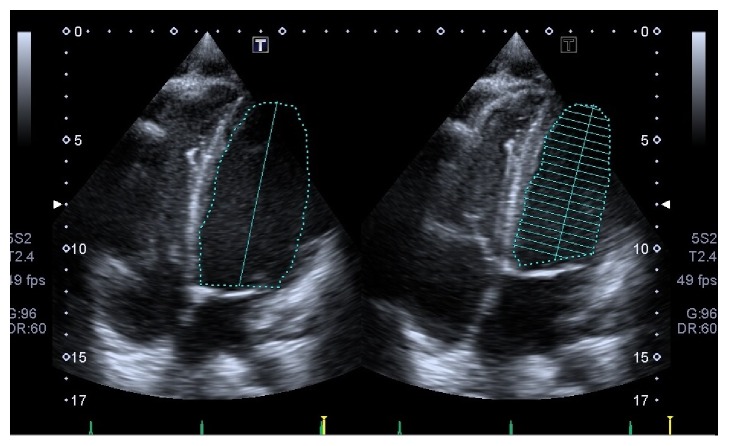
Apical view, apical position showing reduced apical wall motion of both ventricles (left ventricular ejection fraction 45%).

**Figure 3:**
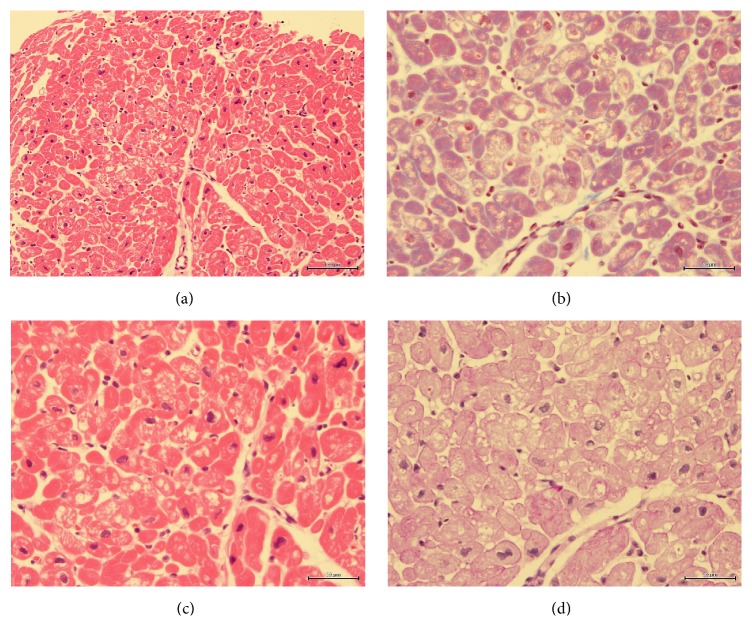
Photographs showing histopathological endomyocardial biopsy of the left ventricle. (a) Hematoxylin and eosin stain; scale bar = 100 *μ*m. (b) Hematoxylin and eosin stain; scale bar = 50 *μ*m. Vacuolar degeneration and hypertrophy of myocardium of LV; no interstitial edema; no inflammatory cell infiltrate. (c) (Masson's trichrome stain) interstitial fibrosis of myocardium. (d) (Periodic acid-Schiff stain) no accumulation of glycogen.

A 17-year-old female with no medical history complained of general fatigue and bradycardia from three months before. Her height, body weight, and body mass index (BMI) were 161 cm, 45 kg, and 17.4 kg/m^2^, respectively. A physical examination indicated that the blood pressure and heart rate are 87/46 mmHg and 38 beats/min, respectively. Laboratory data indicated an elevation of hepatic enzymes (aspartate aminotransferase, 21 IU/L; alanine aminotransferase, 28 IU/L), hypoproteinemia (total protein, 6.1 g/dL; albumin, 3.9 g/dL), hypoglycemia (blood glucose, 67 mg/dL), and a decrease in free triiodothironene (FT3) and free thyroxine (FT4) levels (FT3, 0.87 pg/mL; FT4 0.87 ng/dL). Thyroid stimulating hormone (4.610 *μ*IU/L) was normal. The total blood ketone level was high (114 mmol/L), but the levels of thiamin (2.7 *μ*g/dL), cobalamin (548 pg/mL), folic acid (4.8 ng/mL), growth hormone (6.17 ng/mL), and adrenocorticotropic hormone (31.3 pg/mL) were within normal limits. An electrocardiogram showed bradycardia with junctional rhythm and prolonged QT interval (Figure 1). A cardiac echocardiogram showed reduced left ventricular ejection fraction (LVEF 45%) with apical wall motion decline of both ventricles and both ventricles were dilated (LV diastolic dimension: LVDd 52 mm) (Figure 2). A coronary angiogram showed no stenosis or obstruction, and cardiac magnetic resonance imaging (MRI) did not show late gadolinium enhancement. An endomyocardial biopsy revealed moderate vacuolar degeneration, hypertrophy, and attenuation of the myocardium as well as moderate interstitial fibrosis. Any inflammatory cell infiltrate, myocardial edema, or necrosis was not observed (Figure 3). The patient was diagnosed with idiopathic dilated cardiomyopathy with sick sinus syndrome at the time.

An angiotensin-converting-enzyme inhibitor (perindopril) was prescribed without pacemaker implantation since the fatigue was clearly not related to the bradycardia. During the follow-up, cardiac echocardiographic findings after four months did not improve (LVEF 44%, LVDd 49 mm); her body weight reduced to 34.1 kg (BMI 13.6 kg/m^2^) after losing 16 kg in six months, and she was diagnosed with anorexia nervosa (AN) by a psychiatrist. The patient received nutritional counseling and appropriate psychotherapy to increase her caloric intake. One month later, her body weight increased to 43.5 kg (BMI 16.8 kg/m^2^), heart rate (HR) increased to 66 beats/min with sinus rhythm, and LVEF increased to 55%. One year later, her body weight increased to 54 kg (BMI 20.8 kg/m^2^), HR increased to 70 beats/min, and LVEF rose to 60%.

## 2. Discussion

This is the first report documenting the findings from an endomyocardial biopsy of the LV in an anorexia nervosa patient with sinus bradycardia and left ventricular systolic dysfunction.

The main cardiovascular complications in patients with AN are bradycardia, systolic dysfunction, impaired ventricular repolarization, low blood pressure, QT interval prolongation, mitral valve prolapse, and reduction of myocardial contractility [1, 2]. The mechanistic pathways linking AN to left ventricular systolic function decline are not clear. However, several reports have revealed that cardiomyopathy in patients with AN is in fact a reversible ventricular dysfunction [3]. It is thus believed that patients with AN exhibit conditions involving a decrease in LV systolic function, such as Takotsubo cardiomyopathy, as a result of low blood sugar starvation. Specifically, this case study indicated a decline in the apex LV wall motion that mimics Takotsubo cardiomyopathy. Sachdeva et al. showed that an experimental model of Takotsubo cardiomyopathy presents persistent foci of necrosis and fibrosis [4]. Izgi et al. reported myocardial edema as one of the characteristic features in the pathogenesis of Takotsubo syndrome [5]. Although the patient in our case study showed signs of Takotsubo cardiomyopathy, the endomyocardial biopsy of the LV did not show necrosis and the cardiac MRI did not demonstrate myocardial edema like in Takotsubo cardiomyopathy. As such, the mechanism of left ventricular systolic function might be different from Takotsubo cardiomyopathy.

The LV abnormal wall motion might be caused by energy imbalance, particularly protein-calorie imbalance, from self-induced starvation. Moreover, malnutrition alters the blood concentration of various hormones (e.g., insulin, thyroid hormones, glucocorticoids, and growth hormones). As a result, the myocardium at the cellular level might suffer from diminished protein synthesis, decreased cardiac mass, and changes in myocardial contractility [2]. Consequently, depletion of intramyocardial glycogen and myocardium atrophy could cause a decrease in vagal tone, bradycardia, and QT interval prolongation.

## 3. Conclusions

An anorexia nervosa case with bradycardia and left ventricular systolic dysfunction showed the pathological changes of the myocardium. A girl suspected of having dilated cardiomyopathy needs a differential diagnosis of anorexia nervosa.
